# Efficacy of Intravenous Immunoglobulin/Exchange Transfusion Therapy on Gestational Alloimmune Liver Disease

**DOI:** 10.3389/fped.2021.680730

**Published:** 2021-06-21

**Authors:** Pai-Jui Yeh, Shiu-Feng Huang, Ming-Chou Chiang, Chao-Jan Wang, Ming-Wei Lai

**Affiliations:** ^1^Division of Pediatric Gastroenterology, Department of Pediatrics, Chang Gung Memorial Hospital, Linkou Branch and Chang Gung University College of Medicine, Taoyuan, Taiwan; ^2^Department of Pathology, Chang Gung Memorial Hospital, Linkou Branch and Chang Gung University College of Medicine, Taoyuan, Taiwan; ^3^Institute of Molecular and Genomic Medicine, National Health Research Institutes, Zhunan, Taiwan; ^4^Division of Neonatology, Department of Pediatrics, Chang Gung Memorial Hospital, Linkou Branch and Chang Gung University College of Medicine, Taoyuan, Taiwan; ^5^Department of Medical Imaging and Intervention, Chang Gung Memorial Hospital, Linkou Branch and Chang Gung University College of Medicine, Taoyuan, Taiwan; ^6^Liver Research Center, Chang Gung Memorial Hospital, Linkou Branch, Taoyuan, Taiwan

**Keywords:** gestational alloimmune liver disease, intravenous immunoglobulin, neonatal hemochromatosis, hyperbilirubinemia, neonatal liver failure, exchange transfusion

## Abstract

**Background:** Gestational alloimmune liver disease (GALD) is a rare but critical cause of neonatal liver failure. After discovering the maternal–fetal alloimmune mechanism, intravenous immunoglobulin (IVIG) with or without exchange transfusion (ET) has gradually replaced antioxidant cocktails as the first-line therapy. Whether such therapy changes the outcome of neonates with GALD is yet to be defined.

**Method:** We reported a pair of twins with discordant presentations, mild and self-limited in the older, whereas liver failure in the younger, who was successfully rescued by ET and IVIG. To investigate the outcome after therapeutic alteration, 39 cases between 2005 and 2020 from literature research were collected.

**Results:** Half of the collected cases (47.1%) were preterm. Common presentations were ascites, jaundice, respiratory distress, hepatomegaly, and edema. Leading laboratory abnormalities were coagulopathy, hypoalbuminemia, and elevated serum ferritin. Salivary gland biopsy and magnetic resonance imaging detected extrahepatic siderosis in 70% (14/20) and 56% (14/25), respectively. IVIG, ET, and liver transplantation were performed in 19 (48.7%), 15 (38.5%), and 8 (20.5%) patients, respectively. The overall survival (OS) rate and native liver survival (NLS) rate were 64.1% (25/39) and 43.6% (17/39), respectively. Although the compiled results did not support a significant benefit, the OS and NLS were higher in the IVIG with/without ET group compared with those treated with conventional therapy [OS (70 vs. 57.9%) and NLS (55 vs. 31.6%), respectively].

**Conclusion:** A high index of suspicion for GALD is crucial when facing a neonate with liver failure. Despite no significant influence on the outcome over conventional therapy in such a rare and detrimental disease, IVIG with or without ET can be worth trying before resorting to liver transplantation, which is resource-demanding and technique-challenging in small infants.

## Introduction

Gestational alloimmune liver disease (GALD), currently regarded as a maternal–fetal alloimmune disorder, is a leading etiology of neonatal liver failure and neonatal hemochromatosis (NH) (1). Patients mostly present with congenital cirrhosis, whereas some develop hyperacute process, leading to stillbirth or demise (2). Antenatally, oligohydramnios and intrauterine growth retardation may be noted. Most patients develop clinical signs of liver failure within hours after birth. Laboratory examination may reveal hyperbilirubinemia, coagulopathy, disproportionally normal aminotransferase levels, elevated alpha-fetoprotein, and raised ferritin. Histopathologic and imaging studies are required to confirm extrahepatic siderosis, a fundamental finding for the diagnosis of NH (2). Asides from the classical NH, reports of non-iron-overload cases and novel pathologic features have expanded the spectrum of GALD (2, 3).

Although the causal antigen has not been identified, an *in utero* alloimmune pathway is attributed as early as the 12th week of gestation, inducing specific transplacental immunoglobulin G to activate the complement system and downstream cascades of fetal liver injury (4). High recurrence in the progenies of the affected mother also reflects the successive impact of maternal alloimmunization. In the past two decades, treatment evolution has been elaborated from the perspective of alloimmunization (4–7). Intravenous immunoglobulin (IVIG) and plasma exchange transfusion (ET) have replaced conventional cocktail therapy (1, 2). The application of gestational IVIG also potentially prevents its recurrence in the next pregnancy (2, 8, 9).

The incidence of GALD-NH is around 4 per 100,000 live births in the United States (10). However, the research from Asia seems scanty. We encountered GALD-associated liver failure in a younger twin successfully rescued with ET and IVIG. Meanwhile, another patient whose diagnosis was made until liver transplantation was reported at a local pediatric gastroenterology meeting in Taiwan. Therefore, it is intriguing to understand the effect of IVIG on this rare disease based on a case series collected from a literature review.

## Materials and Methods

### Systematic Review of the Literature

A literature search in the PubMed database using keywords “gestational alloimmune liver disease” and “neonatal hemochromatosis” was conducted, retrieving case reports or case series published in English with full text from January 2005 to March 2020. Case series with only summarized or statistical results were excluded.

Extracted data of interest included countries, gestational ages (GAs) (prematurity defined as GA <37 weeks), sexes, birth bodyweights, modes of delivery, antenatal history (intrauterine growth retardation, oligohydramnios, family history, and other specific prenatal diagnoses), clinical presentations, laboratory results (aminotransferases, bilirubin, coagulation function, albumin, alpha-fetoprotein, and ferritin), magnetic resonance imaging (MRI), histopathologic findings (salivary gland or liver), treatments (ET, IVIG, and liver transplantation), outcomes, and the age of the last follow-up. Elevation of ferritin or alpha-fetoprotein was defined if the value exceeded 800 (ng/ml) and 100,000 (ng/ml), respectively (2). Coagulopathy was defined if the international normalized ratio (INR) exceeded 1.5. Hyperbilirubinemia was defined if total bilirubin exceeded 2 mg/dl. Patients with uncertain diagnoses, history of antenatal IVIG prevention, or fetal death were excluded. A proportion was used in some demographics or laboratory results with missing data. Depiction of differential diagnoses of each report was evaluated in detail. Cases with an evident argument for congenital infection, mitochondrial cytopathy, bile acid synthesis defect, transaldolase deficiency, or syndromic presentation would be excluded.

### Statistical Analyses

Data collection and processing were conducted using Excel and SPSS v. 20 software. Continuous variables were expressed as means ± standard deviations or as medians with the interquartile range as appropriate. Categorical variables were expressed as percentages and were analyzed using the chi-square test. Comparative analyses of descriptive and inferential data were performed using the Student's *t*-test. A *p*-value < 0.05 was considered significant.

## Results

### Our Case

This female neonate was born to a G1P1 mother *via* Cesarean section due to twin pregnancy (dizygotic twin) at a local hospital with GA 37 + 3/7 weeks and a birth bodyweight of 2,115 g without any abnormality in the antenatal examination. The baby required oxygen support for respiratory distress. Abdominal distention was noted at 4 days old. Laboratory test revealed white blood cell 3,980/μl, hemoglobin 10.7 g/dl, platelet 74,000/μl, INR 2.53, albumin 1.9 g/dl, direct/total bilirubin 1.78/4.66 mg/dl, and aspartate transaminase/alanine aminotransferase: 55/22 U/L. She was transferred to our neonatal intensive care unit.

**Figure 1:**
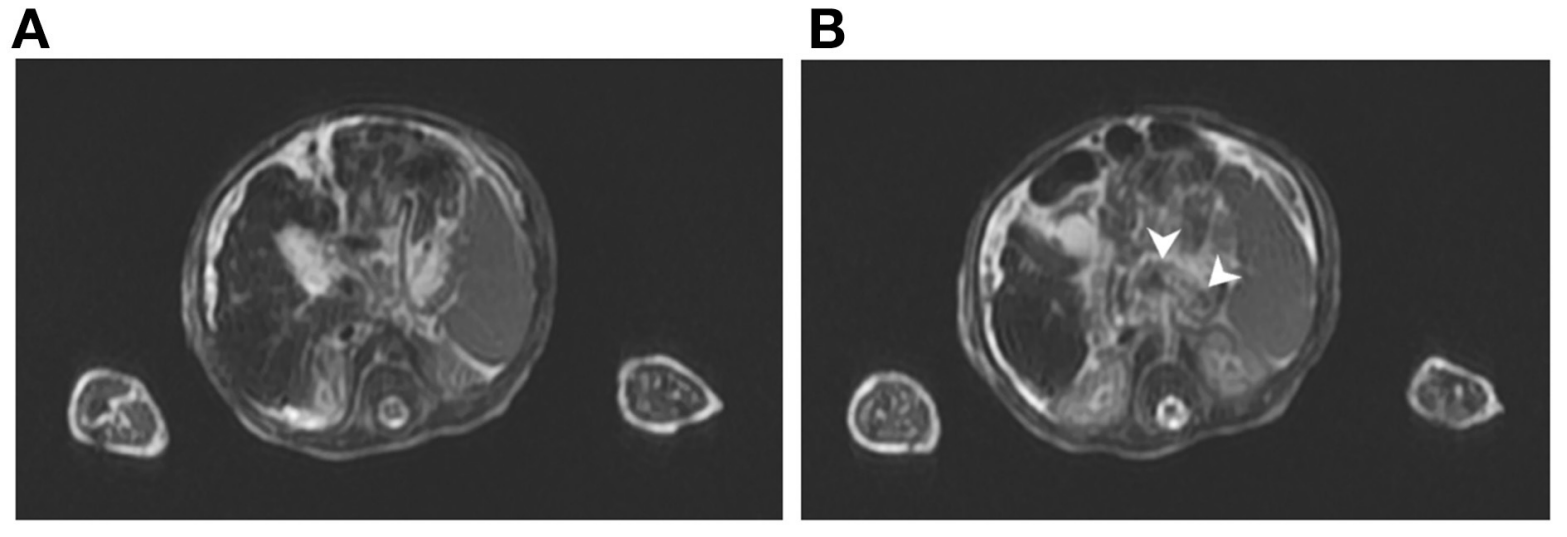
T2-weighted magnetic resonance imaging of case: **(A)** Siderosis and cirrhosis of liver and **(B)** metal-induced susceptibility artifact, indicating siderosis of pancreatic body (arrowhead).

**Figure 2:**
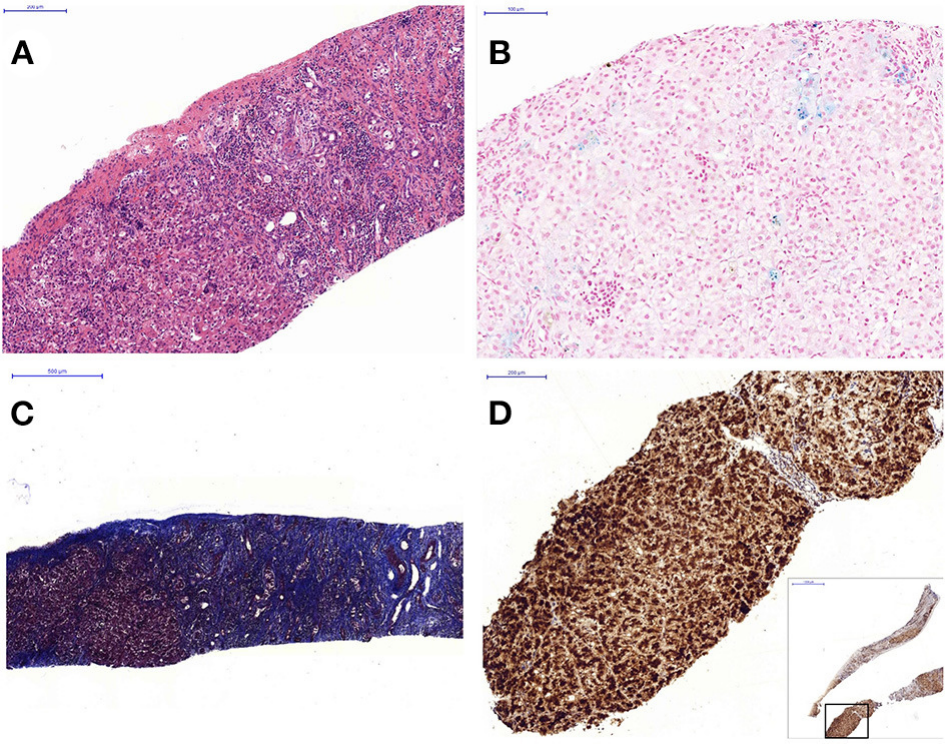
Liver histopathology: **(A)** hematoxylin and eosin stain shows heavy inflammatory cell infiltration including eosinophils and residual hepatic hematopoiesis (100×), **(B)** iron stain shows grade 1 siderosis (blue-stained area) (200×), **(C)** Masson Trichrome stain shows severe fibrosis with cirrhotic change (40×). **(D)** Immunohistochemistry stain with C5b-9 antibody shows strongly positive cells (brownish stained area) involving 90% of the tissue (100×).

The abdominal sonography reported splenomegaly and profuse ascites. Congenital infections and surveys for an inborn error of metabolism were all negative except for a lower carnitine level. Blood, urine, and ascites culture yielded no pathogen. GALD was considered and confirmed by the findings of elevated ferritin (1,010 ng/ml), decreased transferrin (79.9 mg/dl), elevated alpha-fetoprotein (285,834.7 ng/ml), and siderosis of the liver and pancreas by MRI (Figure 1). Double volume ET followed by IVIG (1 g/kg) was completed on the 21st day of life. A liver biopsy performed a month later after the subsidence of coagulopathy revealed cirrhosis and siderosis (grade 1) (Figure 2) and positive immunohistochemical (IHC) stain of C5b-9 (Figure 2). During the subsequent follow-up, bilirubin gradually normalized in 4 months. Fibroscan at 1 year and 9 months old showed a liver stiffness measurement of 8.4 Kpa (F2).

Her dizygotic twin A was born with a birth bodyweight of 2,540 g. Laboratory examination disclosed INR 1.45, aspartate transaminase/alanine aminotransferase 272/43 U/L, direct/total bilirubin 0.95/1.65 mg/dl, albumin 2.2 g/dl, alpha-fetoprotein 97025.8 ng/ml, transferrin 92 mg/dl, and ferritin 762.4 ng/ml. After being transferred to our hospital, repeated laboratory tests did not fulfill the diagnostic levels (ferritin: 358.9 ng/ml, alpha-fetoprotein: 97,025.8 ng/ml). She was kept on an observation plan with continuous improvement.

Because the maternal factors may participate in the pathogenesis, we checked the asymptomatic mother with findings of an elevated serum immunoglobulin G level (2,610 mg/dl), a declined complement level (C3 70.7 mg/dl), positive antinuclear antibody, and positive anti-double-strand DNA antibody.

### Case Series

#### General Demographics

A total of 45 cases from 41 articles were collected from 2005 to 2020. We excluded three cases with antenatal IVIG usage, one with an uncertain diagnosis, one with NH due to mitochondrial long-chain fatty acid oxidation defect, one with Martinez–Frias syndrome, and one fetal death. Together with our twin B, 39 cases were enrolled (Table 1). Most of the patients were reported from the United States. In contrast, Japan and Turkey were two leading countries in Asia.

**Table 1:** Baseline data, presentations, examination results, treatments, and outcomes of the 39 cases.

**No**	**Sex**	**GP**	**GA**	**BBW**	**Deli**	**Major presentation**	**Coag**	**Bil↑**	**Alb↓**	**aFP↑**	**Fer↑**	**MRI**	**Sal**	**L path/Autopsy**	**IVIG**	**ET**	**LT**	**Out**.
1	M	G1P0	35	-	SD	J, E, As, Hm	Y	Y	Y	-	Y	(+) Sid L, P, Adr	N	-	-	-	-	Alive
2	F	G6P2	36	1310	SD	J, R	Y	Y	Y	-	Y	-	Y	-	-	-	-	Alive
3	M	G2P2	33	1843	CS	R, S	Y	Y	-	-	-	-	Y	-	-	-	-	Dead
4	F	G2P2	37	-	SD	As, R, S, Hg	Y	Y	Y	-	Y	(+) Sid L, P, T, Bm	-	Sid, Cir	-	-	Y	Alive
5	M	-	37	-	CS	R, S	-	Y	-	-	-	(+) L	Y	Sid, Cir	-	-	-	Dead
6	M	-	38	-	CS	Hg, IUGR	Y	Y	Y	-	-	(+) Sid L, P, T	-	Sid, Cir	-	-	Y	Alive
7	M	G3P2	37+5	2200	CS	R, As, Hm	Y	Y	Y	Y	Y	(+) Sid L	Y	-	-	-	-	Alive
8	F	-	-	2500	-	J, Hm	Y	Y	-	-	Y	(+) Sid L	Y	-	-	-	Y	Alive
9	F	G1P1	-	1500	-	HG	-	Y	-	-	Y	-	-	Sid	-	-	-	Dead
10	F	G4P2	37	3550	CS	E, As, Hg	Y	Y	Y	-	-	(+) Sid L, P	Y	Sid, Cir	Y	Y	-	Dead
11	M	-	Term	1786	-	Retinal E, As	Y	Y	Y	-	Y	(-) Sid	Y	Sid	-	-	Y	Alive
12	M	G2P1	38	3150	-	J, Hg	Y	Y	Y	Y	Y	(+) Sid L, P	-	Cir	Y	Y	-	Alive
13	F	G4P2	35	2530	-	R, Hg	Y	Y	Y	Y	-	(+) Sid L	Y	-	Y	-	-	Alive
14	-	G4P3	-	3300	CS	J, E, As, Hm	Y	Y	Y	Y	Y	(+) Sid L, P	-	Sid	-	-	-	Dead
15	F	-	-	3600	-	J, E, R, Hm, As	Y	Y	Y	Y	Y	-	-	Sid	-	-	-	Dead
16	F	G2P1	30	1750	CS	HF, R, As	Y	Y	Y	Y	Y	-	-	Sid	Y	-	-	Dead
17	-	G1P1	38	3000	CS	J, E, As	Y	Y	Y	Y	Y	(+) Sid L, P	-	Sid	-	-	-	Dead
18	-	G3P0	37+4	2220	CS	As, Hm	-	Y	-	-	-	(-) Sid, Cir	-	Cir, C5b9	-	-	-	Alive
19	F	G6P4	38+6	2200	SD	J, R, IUGR	Y	Y	Y	-	Y	-	Y	-	Y	Y	-	Alive
20	M	G1P1	37+5	2122	SD	J, Hg, IUGR, Hm, As	Y	Y	Y	-	Y	(-) Sid	N	Sid, Cir, C5b9	Y	Y	Y	Alive
21	M	G3P2	36+6	4345	CS	HF, D, As	Y	-	Y	-	Y	(-) Sid	N	Sid, Cir	Y	-	-	Alive
22	F	G1P1	39	2724	SD	Hg, As	Y	Y	Y	Y	Y	(+) Sid L, P	N	-	Y	Y	-	Alive
23	-	-	Term	3200	-	J, Hg	Y	Y	Y	-	Y	(+) Sid CNS	Y	Sid, Cir	Y	-	Y	Alive
24	F	G3P3	34	1830	CS	Hm, As	Y	-	Y	-	Y	(+) Sid L, P	-	Sid, Cir	-	-	-	Alive
25	F	G2P2	36+2	1270	SD	Hg, Hm, As	Y	Y	Y	-	Y	(+) Sid L	-	Sid, Cir	-	-	Y	Alive
2627 28+	MF F	G1P1G1P1 -	27+6 31+4 32+3	591835 1594	CSCS -	IUGRJ, As, Hm, IUGR Hg	YY Y	YY Y	YY Y	– -	YY Y	-(+) Sid L -	-N -	Sid, Cir, C5b9, ehsSid, Cir, ehs Cir	– Y	Y- Y	– Y	DeadDead Alive
29	F	-	-	-	-	As	Y	-	-	Y	Y	(+) Sid L, P, H, Adr	-	-	Y	Y	-	Alive
30^*^	M	-	37	2490	CS	E, R, Hg, Hm	Y	Y	-	-	Y	(+) Sid L	-	-	Y	-	-	Alive
31^*^	M	-	37	2500	CS	Hm	Y	Y	-	-	Y	(+) Sid L	-	-	-	-	-	Alive
32	M	G3P3	33+4	-	CS	R, E, Hg, Hm	Y	Y	Y	Y	Y	-	Y	-	Y	Y	-	Alive
33	M	G6P4	39+6	-	-	R, Hg	Y	Y	Y	Y	Y	(+) Sid L, P, Bm	N	-	Y	Y	-	Alive
34	F	G3P0	32+2	-	CS	R, E, As	Y	Y	Y	-	Y	(+) Sid L	-	-	Y	Y	-	Alive
35	M	-	35	1600	-	Hg, IUGR	Y	Y	-	-	Y	-	Y	Sid	Y	Y	-	Dead
36	M	-	-	2900	CS	J, R, S, D, Hm, As	Y	Y	Y	-	Y	-	-	Sid, Cir	-	-	-	Dead
37	F	G3P3	35	3115	CS	HF	Y	Y	-	-	Y	(+) Sid L, P	Y	-	Y	Y	-	Dead
38	-	-	37	3200	-	S	Y	Y	-	-	Y	-	Y	Sid, ehs	Y	Y	-	Dead
39^**^	F	G1P1	37+3	2115	CS	R, As	Y	Y	Y	Y	Y	(+) Sid L, P	-	Sid, Cir	Y	Y	-	Alive

*Adr, adrenal; aFP, alpha-fetoprotein; Alb, albumin; As, ascites; BBW, birth bodyweight; Bil, bilirubin; Bm, bone marrow; Cir, cirrhosis or fibrotic change; Coag, coagulopathy; CS, cesarean section; C5b9, positive stain of C5b9; Deli, delivery route; D, Down syndrome-like presentation; E, edema; ehs, extrahepatic siderosis; ET, exchange transfusion; F, female; Fer, ferritin; GA, gestational age; GP, gravidity and parity; H, heart; HF, hydrops fetalis; Hm, hepatomegaly; Hg, hypoglycemia; HG, hyperglycemia; IUGR, intrauterine growth retardation; IVIG, intravenous immunoglobulin; J, jaundice; L, liver; LT, liver transplantation; M, male; Out, outcome; P, pancreas; path, pathology; R, respiratory distress; Sal, salivary gland biopsy; SD, spontaneous delivery; Sid, siderosis; T, thyroid gland*;

* *, monozygotic twins*;

** *, our case; +, family history of GALD*.

The male-to-female ratio was close to 1. Prematurity comprised 47.1% of cases, mostly belonging to the late-preterm (35–37 weeks). Intrauterine growth retardation (IUGR) and oligohydramnios were documented in 10 (25.6%) and 5 (12.8%) cases, respectively. A pair of monozygotic twins (case numbers 30 and 31 in Table 1) was included.

#### Clinical Presentations

The most common presentations were ascites, jaundice, respiratory distress, hypoglycemia, hepatomegaly, and edema (including hydrops fetalis) (Table 2). Down syndrome was observed in three. Several cases developed rare organ dysfunction associated with extrahepatic siderosis, including one with pituitary dysfunction and secondary hypothyroidism and another with bilateral retinal edema. One case developed esophageal varices with hematemesis.

**Table 2:** Major clinical presentation.

**Clinical presentation**	**Number (%)**
Ascites	20 (51.3)
Prematurity	16 (47.1)^a^
Respiratory distress	15 (38.5)
Hepatomegaly	14 (35.9)
Hypoglycemia	13 (33.3)
Jaundice	12 (30.8)
Edema (including hydrops fetalis)	11 (28.2)
Shock/Sepsis-like presentation	8 (20.5)
Patent ductus venosus	5 (12.8)
Hyperglycemia	2 (5.1)
Retinal edema	1 (2.6)

a *Five cases with missing data were excluded*.

#### Laboratory Examination and Imaging Evaluation

Coagulopathy, hyperbilirubinemia, hypoalbuminemia, and elevated ferritin were seen in 100% (36/36), 89.2% (33/37), 96.6% (28/29), and 94.3% (33/35) of patients, respectively. However, alpha-fetoprotein and aminotransferase levels were missed in 66.6 and 30.8% of the cases, respectively.

Twenty-five cases (64.1%, 25/39) received an abdominal MRI. Hepatic siderosis was found in 21 (84%, 21/25), and extrahepatic siderosis in 14 (56%, 14/25), involving the pancreas, the heart, and the adrenal gland. A case with adenohypophysis siderosis had a reversal of iron overload after liver transplantation. *Via* imaging, liver fibrosis or cirrhosis was documented in six (15.4%, 6/39).

#### Histopathological Findings

Lip or buccal mucosa biopsy was performed in 20 cases (51%, 20/39), with 14 (70%, 14/20) positive for iron deposition. Liver tissue was obtained in 24 patients (62%, 24/39, including autopsy in 8) with iron deposition in 21 (87.5%, 21/24). Regarding the three patients without hepatic siderosis, one demonstrated strong C5b-9 IHC staining, another received biopsy 6 months post-ET and IVIG, and the other presented with marked hepatocytes loss and fibrosis. Only three cases applied C5b-9 IHC staining as a diagnostic modality. *Via* histopathology, liver fibrosis or cirrhosis was documented in 16 patients (66.7%, 16/24). If combining MRI and histopathology, siderosis was documented in 37 (94.9%, 37/39) and liver fibrosis/cirrhosis in 18 (46.2%, 18/39).

#### Treatment and Outcome

Conventional cocktail therapy comprising antioxidants, chelating agents, and blood transfusion was applied for the management in the earlier cases. IVIG or ET application became more prevalent after 2010. Seven patients reported before 2010 were all treated “exclusively” with conventional therapy with or without liver transplantation.

IVIG, ET, and liver transplantation were performed in 19 (48.7%), 15 (38.5%), and 8 (20.5%) patients, respectively. Among them, 14 cases (35.9%) received both ET and IVIG. The IVIG dose was 1 g/kg, whereas a few cases received multiple doses up to 5 g/kg in 2 weeks. The administration timing varied as early as the second day of life. As to ET, around 25% of cases underwent multiple courses (33 courses in one). The ET timing usually accompanied or preceded IVIG.

In this series, the overall survival and native liver survival (NLS) rates were 64.1% (25/39) and 43.6% (17/39), respectively. On survival analysis of IVIG with/without ET vs. the conventional cocktail therapy groups, the overall survival and NLS rates were higher in the IVIG with/without ET group, although not statistically significant (Table 3). Hence, a significant benefit was not supported by the current compiled results.

**Table 3:** Survival rates in GALD cases receiving IVIG with/without ET or conventional therapy.

	**IVIG ± ET^†^*N* = 20**	**Conventional^‡^*N* = 19**	P^*^
Overall survival, *n* (%)	14 (70)	11 (57.9)	0.5145
Expired, *n* (%)	6 (30)	8 (42.1)	
Native liver survival, *n* (%)	11 (55)	6 (31.6)	0.2003
Expired + liver transplantation, *n* (%)	9 (45)	13 (68.4)	

* *Statistics by Fisher exact test*.

† *IVIG, intravenous immunoglobulin; ET: exchange transfusion*.

‡ *Conventional therapy: antioxidant cocktails*.

## Discussion

We shared a successful experience of a neonate (the younger twin) with GALD-related liver failure, managed with ET followed by IVIG with rapid clinical improvement, gradual resolution of stigmata of portal hypertension, and regression of liver fibrosis from cirrhosis (F4) to F2 (Fibroscan). Complete regression of cirrhosis has been reported in some cases approximately 2 to 3.5 years later (11).

Interestingly, the twin A of our case presented minor liver dysfunction, echoing the discordant presentations in reported GALD twins (12–14). Similar to different hemolytic severity between twins born to rhesus-sensitized mothers, attributed to alloantigen polymorphism or dissimilar antigen presentation by HLA variants (15), a parallel mechanism may apply to GALD-NH. Dizygosity of our twin may well-explain the different manifestations in siblings born to the same mother.

NH may arise from non-GALD etiologies, including perinatal infection, trisomy 21, mitochondrial DNA depletion due to deoxyguanosine kinase deficiency (DGUOK gene mutations), bile acid synthetic defect (SRD5B1 mutations), GRACILE syndrome (BCS1L mutation), myofibromatosis, tricho-hepato-enteric syndrome, and Martinez–Frias syndrome (2). Although Down syndrome (DS)-associated NH may be explained by transient megakaryocytic leukemia (TML), in some DS with NH, TML was not observed in the pathology or autopsy (16). Maternal antinuclear antibodies were documented at pregnancy in another reported DS-NH case without TML, which supported an immune-mediated mechanism (17). This series included three NH cases associated with DS without TML. Two of them received IVIG therapy with survival in one.

Global incidence, geographic distribution, and ethnic influence remain unknown. Large-scale studies and case series (over five cases) are mostly reported from the United States and Europe, whereas the reports in the Asian population are exiguous (5, 8, 9, 17–23). Underdiagnosis and the lack of a reporting system may lead to scarcity.

The clinical manifestation can be non-specific, resembling neonatal sepsis, respiratory failure, or metabolic disorders. Some presented with mild disease, whereas some suffered from complications of liver cirrhosis or fatal multiorgan failure. The challenge emphasizes the high suspicion of GALD when encountering neonates with acute severe liver disease (9, 17–19).

Prenatal clues of GALD-NH are not common. Sonogram or MRI may reveal oligohydramnios, IUGR, hydrops fetalis, atrophic liver, splenomegaly, iron deposition in liver or pancreas, edematous placenta, or cholelithiasis (24, 25). IUGR (25.6%) and oligohydramnios (12.8%) in this series were in the low end compared with previous studies, ranging from 20.6 to 100% and from 8 to 100%, respectively (17, 18, 20, 23, 24, 26). Still, these two findings are not exclusive to NH.

Extrahepatic siderosis is the fundamental criterion for the diagnosis of NH. However, diagnosis of GALD may still be justified if recurrence in siblings without evidence of extrahepatic siderosis (27). Another substantial pathological evidence is the demonstration of C5b-9 IHC staining, indicating complement-mediated liver injury. This neoantigen produced during the terminal complement cascade activation has been recognized as a defining feature for GALD (28). Cases with intense hepatic C5b-9 staining and supportive clinical presentation but an absence of extrahepatic siderosis have been reported (29). However, the specificity of C5b-9 staining is still arguable. A study of 44 cases with neonatal liver failure (22 NHs, and 22 non-NHs) that were found only six in the NH group were definite GALD if based on C5b-9 IHC (20). Dubruc et al. reported C5b-9 that was expressed in 100% of GALD cases, yet also in 27% of non-GALD cases (enterovirus hepatitis, bile acid synthetic defect, DGUOK mutation, Gaucher disease, cystic fibrosis, and giant-cell hepatitis with autoimmune hemolytic anemia) (30). Regarding these controversial findings, a careful application and interpretation of the C5b-9 IHC are indispensable for an accurate diagnosis.

The diagnosis rate of siderosis by MRI and salivary gland biopsy varied. Isa et al. reported 100 and 37.5% of hepatic and pancreatic siderosis by MRI, respectively; Feldman et al. suggested 60% of sensitivity by either MRI or salivary gland biopsy, whereas the combination of both tools yielded 80% (2, 19). This series showed hepatic siderosis by MRI in 84% of cases. As for extrahepatic siderosis, MRI and salivary gland biopsy detection rates were 56 and 70%, respectively.

Based on the alloimmune mechanism, treatment of GALD has switched from conventional cocktail therapy to the combination of ET and IVIG while saving liver transplantation for refractory cases. ET acts to remove existing reactive antibody, and IVIG blocks antibody-induced complement activation (2). The historically high mortality rate, reaching 80–90%, has been reduced with this revolutionary therapy (2, 4). Nevertheless, the survival rate differed in several reports. A significant difference in NLS between the IVIG/ET groups (75–79%) and the historical conventional therapy group (17%) was documented (9). However, Taylor et al. found a lower NLS of 45% in the IVIG with/without ET group in a different cohort (8). The NLS with IVIG with/without ET in this series (55%) was amid the two reported cohorts. The different outcomes may be multifactorial, including possible alternative diagnoses with NH, disease severity, the timing of application, doses, and single or combo use of ET and IVIG.

## Conclusion

GALD-NH is an important etiology of neonatal liver failure with the insult beginning in the fetal stage. Evidence of extrahepatic siderosis and complement-mediated hepatic injury are supportive features, but more research is required to define the specificity of different diagnostic modalities. Discordant presentations in twins suggest an independent *in utero* alloimmune process. Currently, IVIG with or without ET is recommended as the first-line therapy. Although IVIG/ET therapy did not significantly improve NLS in this series, it is worth trying in a neonate with liver failure before resorting to liver transplantation, which is resource-demanding and technique-challenging in small infants.

## Data Availability Statement

The original contributions presented in the study are included in the article/Supplementary Material, further inquiries can be directed to the corresponding author/s.

## Ethics Statement

Ethical review and approval was not required for the study on human participants in accordance with the local legislation and institutional requirements. Written informed consent to participate in this study was provided by the participants' legal guardian/next of kin.

## Author Contributions

M-WL, P-JY, S-FH, M-CC, and C-JW: substantial contributions to conception and design, acquisition of data, or analysis and interpretation of data. M-WL and P-JY: drafting the article. M-WL: critical revision. All authors: final approval.

## Conflict of Interest

The authors declare that the research was conducted in the absence of any commercial or financial relationships that could be construed as a potential conflict of interest.
