# Supplementary material for: Efficacy of Intravenous Immunoglobulin/Exchange Transfusion Therapy on Gestational Alloimmune Liver Disease
Source: Front Pediatr. 2021 Jun 21;9:680730. doi: 10.3389/fped.2021.680730 (PMC8255369; doi:10.3389/fped.2021.680730)
Supplement: Supplementary file 1 [file Table_1.DOCX]

Supplementary table: References of the case series

| Case | Reference |
| --- | --- |
| 1 | Ekong UD, Kelly S, Whitington PF. Disparate clinical presentation of neonatal hemochromatosis in twins. Pediatrics. 2005;116(6):e880-4. |
| 2 | Chan KC, Edelman M, Fantasia JE. Labial salivary gland involvement in neonatal hemochromatosis: a report of 2 cases and review of literature. Oral Surg Oral Med Oral Pathol Oral Radiol Endod. 2008;106(1):e27-30. |
| 3 | Chan KC, Edelman M, Fantasia JE. Labial salivary gland involvement in neonatal hemochromatosis: a report of 2 cases and review of literature. Oral Surg Oral Med Oral Pathol Oral Radiol Endod. 2008;106(1):e27-30. |
| 4 | Tsai A, Paltiel HJ, Sena LM, Kim HB, Fishman SJ, Alomari AI. Neonatal hemochromatosis and patent ductus venosus: clinical course and diagnostic pitfalls. Pediatr Radiol. 2009;39(8):823-7 |
| 5 | Tsai A, Paltiel HJ, Sena LM, Kim HB, Fishman SJ, Alomari AI. Neonatal hemochromatosis and patent ductus venosus: clinical course and diagnostic pitfalls. Pediatr Radiol. 2009;39(8):823-7 |
| 6 | Tsai A, Paltiel HJ, Sena LM, Kim HB, Fishman SJ, Alomari AI. Neonatal hemochromatosis and patent ductus venosus: clinical course and diagnostic pitfalls. Pediatr Radiol. 2009;39(8):823-7 |
| 7 | Pearson L, Bissinger R, Romero KR. Neonatal hemochromatosis: a case report. Adv Neonatal Care. 2009;9(2):72-6. |
| 8 | Sharma A, Cotterell AH, Maluf DG, Posner MP, Fisher RA. Living donor liver transplantation for neonatal hemochromatosis using non-anatomically resected segments II and III: a case report. J Med Case Rep. 2010;4:372-. |
| 9 | Cetinkaya S, Kunak B, Kara C, Demirceken F, Yarali N, Polat E et al. A case report of neonatal diabetes due to neonatal hemochromatosis. J Pediatr Endocrinol Metab. 2010;23(5):521-4. |
| 10 | Neil E, Cortez J, Joshi A, Bawle EV, Poulik J, Zilberman M et al. Hepatic failure, neonatal hemochromatosis and porto-pulmonary hypertension in a newborn with trisomy 21--a case report. Ital J Pediatr. 2010;36:38-. |
| 11 | Maldonado RS, Freedman SF, Cotten CM, Ferranti JM, Toth CA. Reversible retinal edema in an infant with neonatal hemochromatosis and liver failure. J AAPOS. 2011;15(1):91-3. |
| 12 | Cakir M, Mutlu M, Aydin-Mungan S, Cansu A, Aslan Y, Erduran E. Neonatal hemochromatosis: a case report with unique presentation. Turk J Pediatr. 2011;53(4):455-9. |
| 13 | Magliocca KR, Lewis EL, Bhattacharyya I, Cohen DM, Dixon LR. Labial salivary gland biopsy in the investigation of neonatal hemochromatosis. J Oral Maxillofac Surg. 2011;69(10):2592-4. |
| 14 | Annagür A, Altunhan H, Yüksekkaya HA, Örs R. Therapeutic management of neonatal hemochromatosis: Report of four cases and literature review. Human & Experimental Toxicology. 2011;30(10):1728-34. |
| 15 | Annagür A, Altunhan H, Yüksekkaya HA, Örs R. Therapeutic management of neonatal hemochromatosis: Report of four cases and literature review. Human & Experimental Toxicology. 2011;30(10):1728-34. |
| 16 | Annagür A, Altunhan H, Yüksekkaya HA, Örs R. Therapeutic management of neonatal hemochromatosis: Report of four cases and literature review. Human & Experimental Toxicology. 2011;30(10):1728-34. |
| 17 | Annagür A, Altunhan H, Yüksekkaya HA, Örs R. Therapeutic management of neonatal hemochromatosis: Report of four cases and literature review. Human & Experimental Toxicology. 2011;30(10):1728-34. |
| 18 | Debray FG, de Halleux V, Guidi O, Detrembleur N, Gaillez S, Rausin L et al. Neonatal liver cirrhosis without iron overload caused by gestational alloimmune liver disease. Pediatrics. 2012;129(4):e1076-9. |
| 19 | Babor F, Hadzik B, Stannigel H, Mayatepek E, Hoehn T. Successful management of neonatal hemochromatosis by exchange transfusion and immunoglobulin: a case report. J Perinatol. 2013;33(1):83-5. |
| 20 | Tsunoda T, Inui A, Kawamoto M, Sogo T, Komatsu H, Kasahara M et al. Neonatal liver failure owing to gestational alloimmune liver disease without iron overload. Hepatol Res. 2015;45(5):601-5. |
| 21 | Jimenez-Rivera C, Gupta A, Feberova J, de Nanassy JA, Boland MP. Successful treatment of neonatal hemochromatosis as gestational alloimmune liver disease with intravenous immunoglobulin. J Neonatal Perinatal Med. 2014;7(4):301-4. |
| 22 | Machtei A, Klinger G, Shapiro R, Konen O, Sirota L. Clinical and Imaging Resolution of Neonatal Hemochromatosis following Treatment. Case Rep Crit Care. 2014;2014:650916. |
| 23 | Indolfi G, Berczes R, Pelliccioli I, Bosisio M, Agostinis C, Resti M et al. Neonatal haemochromatosis with reversible pituitary involvement. Transpl Int. 2014;27(8):e76-9. |
| 24 | Korkmaz L, Bastug O, Daar G, Doganay S, Deniz K, Kurtoglu S. Neonatal hemochromatosis in monochorionic twins. J Neonatal Perinatal Med. 2015;8(4):413-6. |
| 25 | Choi SJ, Choi JS, Chun P, Yoo JK, Moon JS, Ko JS et al. Living Related Liver Transplantation in an Infant with Neonatal Hemochromatosis. Pediatr Gastroenterol Hepatol Nutr. 2016;19(2):147-51. |
| 26 | Clarke NE, Gilby D, Savoia H, Oliver MR, Rogerson S. Fulminant liver failure in a neonate. J Paediatr Child Health. 2016;52(3):338-41. |
| 27 | Roos Mariano da Rocha C, Rostirola Guedes R, Kieling CO, Rossato Adami M, Cerski CT, Goncalves Vieira SM. Neonatal Liver Failure and Congenital Cirrhosis due to Gestational Alloimmune Liver Disease: A Case Report and Literature Review. Case Rep Pediatr. 2017;2017:7432859. |
| 28 | Okada N, Sanada Y, Urahashi T, Ihara Y, Yamada N, Hirata Y et al. Rescue case of low birth weight infant with acute hepatic failure. World J Gastroenterol. 2017;23(40):7337-42. |
| 29 | Sokollik C, Kreiter B, Wolf R. Neonatal Hemochromatosis: Blitz Diagnosis Results in Favorable Outcome. J Pediatr. 2017;184:234. |
| 30 | Midorikawa H, Mizuochi T, Okada JI, Hisano T. Disparate clinical findings in monochorionic twins with neonatal hemochromatosis. Pediatr Int. 2017;59(11):1215-6. |
| 31 | Midorikawa H, Mizuochi T, Okada JI, Hisano T. Disparate clinical findings in monochorionic twins with neonatal hemochromatosis. Pediatr Int. 2017;59(11):1215-6. |
| 32 | Kasko O, Klose E, Rama G, Newberry D, Jnah A. Gestational Alloimmune Liver Disease: A Case Study. Neonatal Netw. 2018;37(5):271-80. |
| 33 | Casas-Alba D, Clotet J, Inarejos EJ, Jou C, Fons C, Molera C. Broadening the spectrum of neonatal hemochromatosis. J Matern Fetal Neonatal Med. 2020;33(6):1024-6. |
| 34 | Nair J, Kumar VHS. Liver Failure and Conjugated Hyperbilirubinemia in a Preterm Neonate: Role of Early IVIG and Exchange Transfusion. AJP Rep. 2018;8(2):e95-e8. |
| 35 | Chee YY, Wong SCM, Wong MSR. Heterogeneous Presentation of Neonatal Hemochromatosis in Dichorionic Twins. AJP Rep. 2018;8(4):e332-e4. |
| 36 | M R, Purkait S, Satapathy AK, John J, Patra S, Mitra S. Neonatal hemochromatosis in a newborn with Down syndrome. Fetal Pediatr Pathol. 2020;39(1):62-70. |
| 37 | Hatayama K, Washio Y, Okamura T, Noda T, Tsukahara H. Neonatal hemochromatosis associated with rupture of esophageal varices. Pediatr Int. 2019;61(7):735-7. |
| 38 | Tadros HJ, Gupta D, Childress M, Beasley G, Rubrecht AE, Shenoy A et al. Sub-acute neonatal hemochromatosis in an infant with hypoplastic left heart syndrome on ventricular assist device awaiting transplantation. Pediatr Transplant. 2019;23(7):e13567. |
